# Pre-Clinical Development of a Recombinant, Replication-Competent Adenovirus Serotype 4 Vector Vaccine Expressing HIV-1 Envelope 1086 Clade C

**DOI:** 10.1371/journal.pone.0082380

**Published:** 2013-12-03

**Authors:** Jeff Alexander, Jason Mendy, Lo Vang, Jenny B. Avanzini, Fermin Garduno, Darly J. Manayani, Glenn Ishioka, Peggy Farness, Li-Hua Ping, Ronald Swanstrom, Robert Parks, Hua-Xin Liao, Barton F. Haynes, David C. Montefiori, Celia LaBranche, Jonathan Smith, Marc Gurwith, Tim Mayall

**Affiliations:** 1 PaxVax Inc, San Diego, California, United States of America; 2 UNC Center for AIDS Research, University of North Carolina at Chapel Hill, Chapel Hill, North Carolina, United States of America; 3 Duke Human Vaccine Institute, Duke University School of Medicine, Durham, North Carolina, United States of America; 4 Department of Surgery, Duke University School of Medicine, Durham, North Carolina, United States of America; Federal University of São Paulo, Brazil

## Abstract

**Background:**

There is a well-acknowledged need for an effective AIDS vaccine that protects against HIV-1 infection or limits *in* *vivo* viral replication. The objective of these studies is to develop a replication-competent, vaccine vector based on the adenovirus serotype 4 (Ad4) virus expressing HIV-1 envelope (Env) 1086 clade C glycoprotein. Ad4 recombinant vectors expressing Env gp160 (Ad4Env160), Env gp140 (Ad4Env140), and Env gp120 (Ad4Env120) were evaluated.

**Methods:**

The recombinant Ad4 vectors were generated with a full deletion of the E3 region of Ad4 to accommodate the *env* gene sequences. The vaccine candidates were assessed in vitro following infection of A549 cells for Env-specific protein expression and for posttranslational transport to the cell surface as monitored by the binding of broadly neutralizing antibodies (bNAbs). The capacity of the Ad4Env vaccines to induce humoral immunity was evaluated in rabbits for Env gp140 and V1V2-specific binding antibodies, and HIV-1 pseudovirus neutralization. Mice immunized with the Ad4Env160 vaccine were assessed for IFNγ T cell responses specific for overlapping Env peptide sets.

**Results:**

Robust Env protein expression was confirmed by western blot analysis and recognition of cell surface Env gp160 by multiple bNAbs. Ad4Env vaccines induced humoral immune responses in rabbits that recognized Env 1086 gp140 and V1V2 polypeptide sequences derived from 1086 clade C, A244 clade AE, and gp70 V1V2 CASE A2 clade B fusion protein. The immune sera efficiently neutralized tier 1 clade C pseudovirus MW965.26 and neutralized the homologous and heterologous tier 2 pseudoviruses to a lesser extent. Env-specific T cell responses were also induced in mice following Ad4Env160 vector immunization.

**Conclusions:**

The Ad4Env vaccine vectors express high levels of Env glycoprotein and induce both Env-specific humoral and cellular immunity thus supporting further development of this new Ad4 HIV-1 Env vaccine platform in Phase 1 clinical trials.

## Introduction

The development of an effective AIDS vaccine has encountered significant barriers including lack of predictive animal models and absence of well-defined correlates of protection [1,2]. Of major concern is the failure of four large efficacy trials, two based on the use of a recombinant HIV-1 Env gp120 (AIDSVAX), a third (“Step” study) based on the use of a replication-deficient Ad5 vaccine vectors [3-5], and a fourth, the HVTN 505 trial using a multiclade DNA prime immunization followed by a replication-deficient multiclade Ad5 boost immunization [6,7]. However, the results of the RV144 ALVAC/AIDSVAX Phase 2b efficacy trial in Thailand showed an estimated efficacy of 31.2% and suggested that a vaccine to prevent HIV-1 infection may be closer than previously thought [1,2,5,8]. However, efficacy was considered modest and insufficient for the vaccine to be implemented as a public health measure [9]. Furthermore, the vaccine had no effect on modifying viral load or CD4^+^ T cell counts in vaccinated individuals who became infected. The vaccine components used in the RV144 trial were administered using a heterologous prime-boost approach. The priming vaccine was a recombinant canarypox vector virus (ALVAC), which is replication-incompetent in humans, expressing Gag, protease and clade E Env gp120 linked to the transmembrane anchoring portion of gp41. The boosting vaccine was the same AIDSVAX B/E gp120 used previously in the AIDSVAX trial in Thailand [5]. Cellular responses were tested in a subgroup of vaccinees with only minimal level of responses observed. Subsequent analyses have revealed potential immune correlates of protection including: 1) V1V2 binding antibodies and 2) CD4^+^ T cell responses targeting epitopes within the V2 region [10,11]. Thus, vaccines designed to induce significant levels of Env gp120-specific V1V2 antibodies and T cell responses may have improved efficacy against HIV-1 infection. Additionally, several studies have suggested that a more robust induction of bNAbs may increase vaccine efficacy and duration. Many viral vaccines rely on the induction of bNAbs as the primary correlate of protection [12]. Specifically, for HIV-1, passive transfer of bNAbs can completely block infection by chimeric SHIV in non-human primates (NHP) studies [13-16]. The potential of bNAbs to protect against HIV-1 infections is also demonstrated by gene-based antibody delivery in humanized mice and NHPs [17,18].

The recent Phase 2b trials of HIV-1 vaccines support a prime-boost approach and the inclusion of a HIV-1 Env glycoprotein. The lack of efficacy in the AIDSVAX trials, VAX004 and VAX003, suggest a need for greater coverage of neutralizing antibody and T cell immunity [4,19-22]. The Step and HVTN 505 trials suggest a need for higher or qualitatively different T cell responses and a need for an Env antigen (Step) that induces robust Env-specific antibody responses (HVTN 505). The RV144 trial which employed a poxvirus vector (both T and B cell immunogens) prime immunization followed by Env glycoprotein boost immunization appeared to provide ‘some’ low but significant protection against HIV-1 infection.

A concern regarding the possibility of vaccine-induced enhancement of acquisition of HIV-1 infection also arose out of the Step trial, since it was confounded by the observation that there were more HIV-1 infections in the vaccine group than the placebo group, an unanticipated result [3,23]. The apparent increase in HIV-1 infections was observed mainly in men, who were either uncircumcised or who had pre-existing Ad5 neutralizing antibody or both. At the time of the interim analysis of the Step trial, enrollment in an analogous study (Phambili) in South Africa with the same vaccine was terminated. Recently, a long term follow-up of the Phambili study suggested a possible, but not significant increase in HIV-1 infections in the vaccine group compared to the placebo [6], but, in this case, there was no relationship to pre-existing Ad5 neutralizing antibody titers, or to the sex of the vaccine recipient. Recently, interim results of the HVTN 505 trial also indicated there was a non-statistically significant increase in the number of HIV-1 infections among volunteers in the investigational vaccine group compared to the placebo group, though, this was not related to Ad5 pre-existing immunity (all participants were required to by Ad5 seronegative at enrollment) [7]. Although all three of these studies included a recombinant Ad5 vector in the vaccine regimen, in the HVTN 505 study, the non-statistically significant increase in HIV-1 infections in the vaccine group began to emerge, after the three DNA priming vaccines, but before the Ad5 booster vaccine, suggesting that something other than pre-existing Ad5 immunity or an Ad5 vector as a specific risk factor(s) for potential enhancement. Two of the three studies demonstrating possible enhancement (Step, Phambili) did not include an Env antigen. In the third, HVTN 505, transgenes specific for Env were included, but unlike RV144 there was no Env glycoprotein boost, and antibody titers, particularly neutralizing antibody to tier 1 viruses, were low or not detected.

One could speculate in the Step, Phambili, and HVTN 505 trials that vaccine induction of activated immune cells may increase the targets for HIV-1 infection. By this rationale, any vaccination or unrelated infection may also increase the numbers of potential targets and enhance a subject’s susceptibility to HIV-1 infection. There may be opposing mechanisms at work following vaccine administration; 1) an increase of immune cell targets which may augment susceptibility to HIV-1 infection; and 2) immune responses induced that neutralize the virus and/or eliminate the virally-infected cell thereby preventing or controlling infection.

The suggestion of enhancement of HIV-1 virus transmission in these trials in the presence of minimal Env-specific antibody responses suggests the need for much more potent vaccine induction of protective antibody responses. One approach may be a replicating vector system to generate Env antigen persistence and thus potentially leading to higher levels of somatic mutations to generate broad neutralizing antibodies [24].

The objective of this study is to build upon two observations. The first is from the RV144 Thai trial, which pointed to heterologous prime-boost as a potentially successful HIV-1 immunization approach. The second is from our recent Phase 1 clinical trial with an orally administered, replication-competent Ad4 vectored vaccine for avian (H5) influenza, in which very high seroprotection and seroconversion were observed with oral Ad4-H5HA prime immunization followed by parenteral inactivated H5HA protein boost immunization [25].

To this end, we have developed HIV-1 candidate vaccines based on Ad4 recombinant vectors expressing HIV-1 1086 clade C full-length (gp160) Env [26], which may provide a means to express and present on the cell membrane the correct conformation of Env appropriate for immunogenicity. This vaccine was compared with other Ad4Env vectors expressing gp140 or gp120 which will be secreted from recombinant vector infected cells. The vaccine was analyzed in vitro following infection of A549 cells for Env-specific protein expression and for recognition of cell surface Env by broadly neutralizing antibodies (bNAbs). Furthermore, the relative capacity of the three Ad4Env constructs to induce Env-specific humoral immunity in small animals was evaluated. Env-specific cellular immunity was evaluated for the Ad4Env160 vector.

The aim of this study was to evaluate recombinant Ad4 vectors encoding HIV *env* 1086 clade C transgene for Env glycoprotein expression and capacity to induce Env-specific immune responses in small animals. These data support further development of the Ad4 HIV-1 Env vaccine platform in Phase 1 clinical trials.

## Materials and Methods

### Ethics Statement

All animal procedures were performed at Explora BioLabs, San Diego, CA. These facilities are accredited by the American Association for the Accreditation for Laboratory Animal Care (AAALAC) and carry appropriate US government assurances (NIH Assurance #A4487-01, USDA #93-R-0512). Explora conducts an IACUC review for all proposed animal studies and approved these studies (rabbit protocol #EB10-033-001, mouse protocol #EB12-030-001). Studies were conducted in accordance with the NIH “Guide for the Care and Use of Laboratory Animals”. Rabbits were allowed to acclimate to the environment for 7 days and mice for 3 days prior to initiation of the studies.

### Cell Lines

A549 cells were used as a cell substrate for the generation and growth of the Ad4Env recombinant viruses. The A549 cell line is a human epithelial lung carcinoma cell line obtained from ATCC #CCL-185, Manassas, VA [27]. An A549 master cell bank has been produced at PaxVax for GMP manufacturing and its potential use as a substrate for vaccines was reviewed at the September 19, 2012 FDA Advisory Committee on Vaccines and Related Biologicals Products (VRBPAC) meeting, where “The committee agreed that there are concerns with human tumor-derived cell lines but that CBER had addressed these safety concerns based upon the currently recommended assays and potential applications of new technologies” [28].

### Construction of Ad4Env Recombinant Viruses

**Figure 1:**
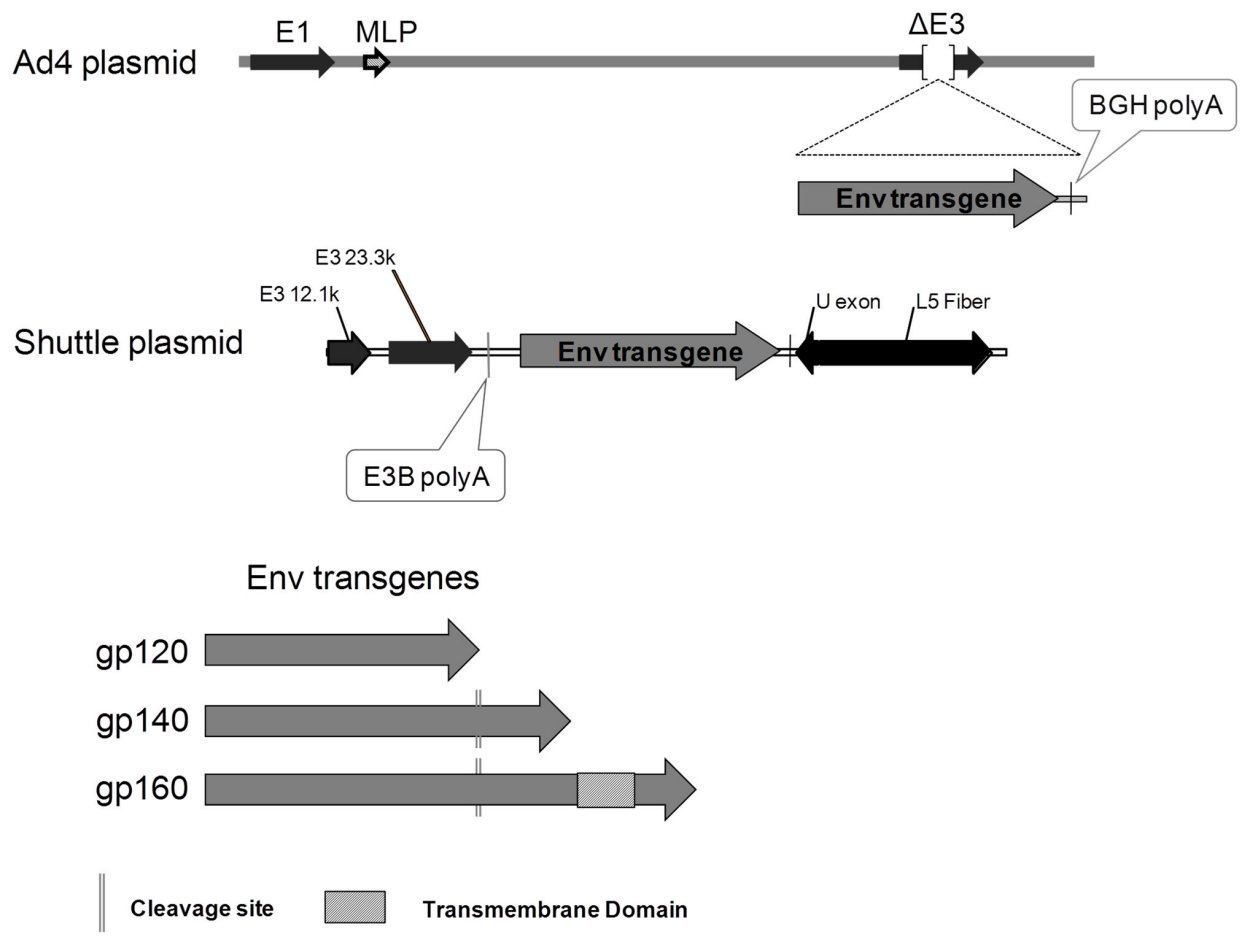
Ad4Env vector design. The HIV-1 env gene sequence was derived from 1086 clade C and inserted into the Ad4 virus E3 region. The use of a shuttle plasmid encoding the Env sequence and the Ad4 plasmid to obtain the final vaccine product is described in Materials and Methods.

Recombinant Ad4-HIV-1-Env viral plasmids were generated by homologous recombination in *E. coli* using a large adenoviral plasmid and a shuttle plasmid containing env transgene and flanking Ad4 sequences as previously described [29]. All *env* genes were derived from the 1086 clade C isolate of HIV-1 [26] and inserted such that codon-optimized env transgene expression is driven by the native adenoviral major late promoter with an added BGH poly-adenylation signal sequence (Figure **1**). The following amino acid sequence AK**R**RVVEREK**R** was mutated to AK**E**RVVEREK**E** to prevent cleavage by furin to gp120 and gp41 glycoproteins. Homologous recombination and identification of correct clones were done as described previously [29]. Recombinant replication competent viruses were generated by transfecting PacI-linearized Ad4Env plasmid DNA into A549 cells. One day before transfection, 2 × 10^6^ A549 cells were plated into 100 mm cell culture dishes (BD Falcon, Franklin Lakes, NJ) in DMEM (Hyclone, Logan, UT) /10% FBS (Hyclone) and the plates were incubated at 37 °C in a humidified atmosphere of 5% CO_2_. The following day, cells at 70-80% confluence were transfected with 10 µg of linearized Ad4Env DNA using Fugene HD transfection reagent as per the manufacturer’s instructions (Roche Applied Sciences, Indianapolis, IN). Two to three days post-transfection, when cells had reached confluence, the A549 cells were expanded into T-series flasks and incubated at 37 °C in a humidified atmosphere of 5% CO_2_ until cytopathic effect (CPE) was observed. Once CPE was observed, generally 7-10 days after transfection, infected cells were harvested and virus was released using 3 freeze-thaw cycles. The lysate was clarified by centrifugation at 1,800 × g for 10 min at 4 °C, and supernatant was collected and the titer estimated by AE-HPLC [30]. Titered virus was subsequently used for testing of Env protein expression by western blot analysis and for scale-up in cellSTACKS® (Corning, Corning, NY).

### Site-Directed Mutagenesis to Generate Env160K→N

The shuttle plasmid containing wild-type *env*160 transgene and flanking Ad4 sequences was used as a template for site directed mutagenesis per manufacturer’s instructions (Quick Change II XL kit, Agilent Technologies, San Diego, CA). Briefly, forward and reverse primers were designed with mutated nucleotides and used to amplify the template by PCR. The template plasmid was digested with *Dpn I* restriction endonuclease leaving the newly synthesized unmethylated nicked PCR product. The product was transformed into *E.coli* cells, where the nick was ligated with host repair enzymes. The resultant plasmid was used in homologous recombination to produce the pPV-Ad4 Env160K→N plasmid.

### Western Analysis to Determine Env Transgene Protein Expression

A549 cells were infected with 5 × 10^8^ vp/mL of Ad4Env120, Ad4Env140 and Ad4Env160 recombinant vectors. After two to three day incubation, the cells were resuspended in RIPA buffer (Thermo Scientific, USA) supplemented with protease inhibitors. The cell lysates were mixed with sodium dodecyl sulfate (SDS) buffer (125 mM Tris-HCl, pH 6.8; 4% SDS; 20% glycerol; 0.01% bromophenol blue; and 10% beta-mercaptoethanol) and boiled for 10 minutes. Cell lysates were then loaded onto a 4-10% polyacrylamide gel. Proteins were transferred to nitrocellulose membrane using iBlot gel transfer device (Life Technologies, USA). The primary detection antibody was the HIV-1 clade C VRC-C 3B3 mAb (from Dr. Barton Haynes, [31]). A horseradish peroxidase (HRP)-conjugated anti-mouse IgG Ab (Southern Biotech, USA) was used as the secondary antibody. The blots were developed using the SuperSignal West Femto blotting detection system (Thermo Scientific, USA).

### FACS Staining of Cell Surface Env gp160 Using bNAbs

A549 cells were harvested using 0.25% Trypsin-EDTA from 80-90% confluent T225 flasks and 25 × 10^6^ cells infected in suspension with 5 × 10^8^ vp/mL of purified Ad4Env160 or Ad4Env160K→N recombinant virus in 5 mL growth medium (Dulbecco’s minimal essential medium, high-glucose (DMEM) containing 10% FBS) for 1 hour at 37 °C with occasional shaking. The 5 mL suspension was then added to 35 mL growth medium, transferred to a T225 flask, and incubated overnight at 37 °C. Eighteen hours after infection, the medium was removed and A549 cells expressing Env160 or Env160K→N glycoprotein were harvested by adding 15 mL FACS buffer [phosphate-buffered saline (PBS), 1% FBS], scraping the monolayer with a cell scraper, and the cell suspension transferred to a 50 mL conical tube followed by centrifugation at 1,200 rpm for 5 minutes to pellet. The cell pellet was resuspended once in FACS buffer to wash, centrifuged, and then resuspended in 5 mL blocking buffer (10% normal goat serum) and incubated on ice for 30 minutes with occasional shaking. The cells were washed again in FACS buffer and stained with dilutions of primary monoclonal HIV-1 Env-specific bNAb at 10, 1, 0.1, and 0.01 µg/mL for 30 minutes at 4 °C. Cells were washed multiple times to remove unbound antibody and incubated with a 1:100 dilution of R-PE-goat anti-human IgG F(ab’)_2_ antibody (Jackson ImmunoResearch, West Grove, PA) for 30 minutes at 4 °C. The cells were washed again thoroughly, fixed with 4% paraformaldehyde (BD Cytofix, BD Bioscience, San Jose, CA) for 30 minutes, then acquired on an Accuri flow cytometer (BD Biosciences). Data were analyzed using FlowJo analysis software (Treestar Inc., Ashland, OR). The bNAbs were kindly provided from several sources which are indicated in the Acknowledgment section.

### Ad4Env120, Ad4Env140, and Ad4Env160 Purified Virus Production for Animal Studies

Expansion of the recombinant viruses was accomplished using 10-Chamber cellSTACKS®. A549 cells were seeded (1 × 10^8^ cells/cellSTACKS®) in culture medium. Cells were infected when 80 to 90% confluence was achieved with Ad4Env120, Ad4Env140, or Ad4Env160 viruses at a concentration of 5 × 10^8^ vp/mL. CellSTACKS® containing the infected cells were incubated at 37 °C and 5% CO_2_ until development of 70-80% CPE, typically after 2 to 3 days incubation. The cells were removed in lysis buffer, and the expanded virus purified from the lysate, following clarification, by anion exchange chromatography and ultrafiltration. Virus identity was confirmed by sequencing across the insertion point and western blot analysis with the concentration of virus particles determined by AE-HPLC [30]. Purified virus was recovered in a Tris-glycerol formulation buffer and stored at −80 °C.

### Immunization Protocol

Female New Zealand White rabbits with body weight of 2 to 4 kg were purchased from Charles River, Wilmington, MA, and housed in the vivarium facilities at Explora BioLabs, San Diego, CA. Rabbits were allowed to acclimate to the environment for 7 days prior to initiation of the study and randomized into 5 immunization groups of 3 rabbits per group. Two Ad4Env vaccine immunizations were given with a one month interval followed by a recombinant Env gp140 boost immunization two months following the second vector immunization. Specifically, rabbits were immunized on days 0 and 28 by the intramuscular (i.m.) route with 1 × 10^11^ vp of Ad4Env160, Ad4Env140, or Ad4Env120 vectors into the quadriceps muscles, with 0.5 mL delivered in each hind leg (1 mL total volume per dose). It should be noted that one group of rabbits was also immunized by the intranasal (i.n.) route with 1 × 10^11^ vp of Ad4Env140 inoculated into each nare in a 75 µL volume using a pipetman (150 µL total volume per dose). An Ad4Env vaccine dose titration was not performed to determine the lowest dose that was sufficient for inducing transgene-specific immune responses. Instead, high doses of vaccine were used to increase the likelihood that immune responses would be generated and could be assessed. Recombinant Env 1086 clade C gp140 (100 µg) formulated in Rehydragel® (General Chemical, Parsippany, NJ) as the booster immunization was given on day 84 by the i.m. route in a total volume of 1 mL. As a negative control, rabbits were immunized three times with 1 × 10^11^ vp of Ad4 wild type (Ad4wt) virus using the same schedule, i.e., days 0, 28, and 84. As the positive control, 100 µg recombinant Env 1086 clade C gp140 formulated in Rehydragel® was given three times using the same schedule. Blood was collected for Env-specific antibody evaluation prior to immunization and following each immunization; days 0, 28, 84, and 112. 

Female C57BL/6 × BALB/c (CB6F1) mice were obtained from Charles River for vaccine immunizations to evaluate vaccine induction of Env-specific T cell responses. Three groups, 6 mice per group, were housed in the vivarium facilities at Explora BioLabs. Mice were allowed to acclimate to the environment for 3 days prior to initiation of the study. Mice were immunized two times on days 0 and 28 with Ad4Env160K→N (lysine to asparagine modification at position 160 in V2 loop), Ad4wt, or recombinant Env 1086 clade C gp140 formulated in MPL/10%Rehydragel® (MPL from InvivoGen, San Diego, 12.5 µg per dose). Specifically, group 1 mice were immunized with 1 × 10^10^ vp of Ad4Env160K→N in 100 µL by the i.m. route (50 µL per hind limb). An Ad4Env vaccine dose titration was not performed to determine the lowest dose that was sufficient for inducing transgene-specific immune responses. Instead, high doses of vaccine were used to increase the likelihood that immune responses would be generated and could be assessed. Group 2 mice were immunized subcutaneously (s.c.) at the base of the tail with 10 µg recombinant gp140 in MPL/10% Rehydragel®, and group 3 immunized i.m. with 1 × 10^10^ vp of Ad4wt virus. On day 56, spleens were isolated for use in the IFNγ ELISPOT assay.

### Splenocyte Isolation and T Cell Depletion

Four weeks following the second immunization, mice from each group were sacrificed, spleens isolated, and splenocytes prepared for use in the IFNγ ELISPOT assay. Briefly, spleens were isolated by dissection and placed in 5 mL PRMI-1640 medium containing 10% FBS (R10) and 50 U/mL benzonase (EMD Biosciences, San Diego, CA). Spleens were expressed through a 70 micron cell strainer, washed, centrifuged, and cells resuspended in 1 × RBC lysis buffer (eBioscience, San Diego, CA). After 5 minutes, the cells were washed with R10-Benzonase, centrifuged, and splenocytes resuspended in 10 mL R10 medium and counted. Whole splenocytes were resuspended at a final concentration of 4 × 10^6^ cells/mL. For CD4^+^ or CD8^+^ T cell depletion, Dynabeads Mouse CD4^+^ or Mouse CD8^+^ (Lyt 2) (Life Technologies, Carlsbad, CA) were used according to the manufacturer’s specifications. Briefly, a fraction of whole splenocytes were centrifuged, medium removed, and cell pellets resuspended in Dynabead Isolation Buffer. Pre-washed resuspended Dynabeads, either CD4^+^ or CD8^+^, were added and incubated with the cells for 30 minutes at 4 °C on a rotator. The mixture was then placed in a magnet for 2 minutes. Supernatants were transferred to a new tube and the cells counted. The depleted splenocytes were then centrifuged to remove the Isolation Buffer, and resuspended in R10 medium at final concentration of 4 × 10^6^ cells/mL.

### Env-Specific ELISA Assays

#### Env gp140 ELISA

High-binding EIA/RIA 92-well microplates were coated overnight at 4 °C with 100 µL 1 µg/mL recombinant Env 1086 clade C gp140 antigen. The plates were washed with 200 µL PBS and blocked for 2 hours at room temperature (RT) using 200 µL 10% normal goat serum (Life Technologies, Carlsbad, CA). After washing, serial 1:3 dilutions of immune serum (100 µL/well) diluted using 10% normal goat serum were added to the plates and incubated for 2 hours at RT. The plates were washed again and wells incubated with a 1:10,000 dilution of horseradish peroxidase (HRP)-conjugated goat anti-rabbit IgG (γ-chain specific) secondary detection antibody (Bethyl Laboratories, Montgomery, TX) for 1 hour at RT. For detection of antigen-specific signal, the plates were washed and 100 µL of 3’, 3’ tetramethylbenzidine (TMB single solution, Life Technologies) added to each well. Color was allowed to develop for 5 minutes then 100 µL 1N HCl added to stop the reaction. Microplates were read at 450 nm on a M3 SpectroMax plate reader (Molecular Devices, Sunnyvale, CA). Data was analyzed using GraphPad Prism analysis software.

#### Env V1V2 ELISA Assays

The gp70 V1V2 CASE A2 clade B fusion protein was used as a coating antigen consists of 96 amino acids of the V1V2 loop domain together with flanking conserved sequences, joined at the C-terminus of a fragment consisting of the first 263 amino acids of the murine leukemia virus (MLV) gp70 protein [32]. AE.A244 and 1086 V1V2 Avi Tag polypeptides were used for clades E and clade C coating antigens, respectively. V1V2 Avi Tag polypeptides were produced as described [33,34]. ELISA plates, 384 well (Corning Costar), were coated with 50 µL of 2 µg/mL antigen in 0.1 M sodium bicarbonate for 1 hour and then blocked with assay diluent [PBS containing 4% (w/v) whey protein/15% Normal Goat Serum, 0.5% Tween-20, and 0.05% Sodium Azide]. Immune sera were incubated for 90 minutes in three-fold serial dilutions beginning at 1:50 followed by washing with PBS/0.1% Tween-20. AP-conjugated goat anti-rabbit secondary antibody (Sigma) was incubated at 1:3,000 for 1 hour in 30 µL, then washed and detected with 30 µL of CBC buffer with 2 mM MgCl_2_ and 1 mg/mL p-NPP [4-Nitrophenyl phosphate di(2-amino-2-ethyl-1,3-propanediol) salt]. Plates were read at 405 nm after 45 minutes.

### HIV-1 Pseudovirus Neutralization in TZM.bl and A3R5.7 Cells

Virus neutralization was measured using a luciferase-based assay in TZM.bl or A3R5.7 cells as previously described [35,36]. Specially, the TZM.bl cell assay was used for evaluating neutralization of HIV-1 tier 1 pseudovirus MW965.26. The A3R5.7 assay was used for the tier 2 IMC.LucR viruses Ce1086_B2.LucR.T2A.ecto, Du151.2.LucR.T2A.ecto, Ce2010_F5.LucR.T2A.ecto, Ce1176_A3.LucR.T2A.ecto, and Du422.1.LucR.T2A.ecto. The assay measures the reduction in luciferase reporter gene expression in TZM.bl following a 48-hour incubation period with a single round of virus infection, and in A3R5.7 cells following limited rounds of replication over a 4-day incubation period. In both assays, the 50% inhibitory dose (ID_50_) titer was calculated as the serum dilution that caused a 50% reduction in relative luminescence units (RLU) compared to the level in the virus control wells after subtraction of cell control RLU. Calculations were performed using a validated macro employing a point-based algorithm with linear interpolation between the two replicates on either side of 50% RLU reduction.

### Measurement of Env-Specific T Cell Responses with an IFNγ ELISPOT Assay

T cell responses specific for 15-mer peptides from the HIV-1 Consensus clade C Env gp160, the recombinant Env gp140 1086 clade C, and heat-inactivated Ad4 wild type virus (72 °C for 1 hour) were evaluated using an IFNγ ELISPOT assay. The complete set of overlapping peptides was obtained from the NIH AIDS Reagent Program (Catalog Number 9499). Peptides, dissolved in dimethyl sulfoxide at a concentration of 20 mg/mL, were combined in sequential order from N- to C-terminus to generate 8 pools of approximately 25 peptides each. For the assay, 96-well assay plates (MSIPS4510, Millipore, Bedford, MA) were coated with 100 µL of 10 µg/mL monoclonal antibody specific for murine IFNγ (clone AN18, Mabtech, Stockholm) by incubation overnight at 4 °C. The plates were washed with PBS and blocked with R10 medium for 1 hour at 37 °C. Erythrocyte-free mouse splenocytes (4 × 10^5^ per well) were added in triplicate to wells containing either no antigen, each peptide pool at a final concentration of 3 µg/mL/peptide, 10 µg/mL of Env gp140, or 9 × 10^8^ vp/well of heat inactivated Ad4wt virus in a total volume of 0.2 mL. Plates were incubated for 24 hours at 37 °C. Wells were washed with PBS followed by 1 hour incubation at RT with 100 µL of 1 µg/mL biotin-conjugated monoclonal antibody specific for murine IFNγ (clone R4-6A2, Mabtech) diluted in PBS containing 10% FBS. Plates were washed again and 100 µL of streptavidin-peroxidase complex (Mabtech) added to each well. After 1 hour incubation at RT, the plates were washed thoroughly with PBS followed by the addition of 100 µL AEC peroxidase substrate solution (Vector Laboratories Inc., Burlingame, CA) for spot development. The reaction was stopped after 5 minutes by washing the plate wells with water. Spots were counted using a CTL S5 Core plate reader (Cellular Technologies Ltd., Shaker Heights, OH) and the data recorded as the number of spot forming cells (SFC) per 1 × 10^6^ splenocytes ± the standard deviation.

### Statistics

#### ELISA

ED_50_ titers for ELISA were calculated as the 50% endpoint response following analysis of the dilution curve using a 4-parameter logistic curve-fit model. The Student t test was used to determine significance between groups. A *p* value of ≤ 0.05 was considered significant using unpaired two-tailed analysis parameters. Calculations and extrapolation of titration curves were performed using GraphPad Prism analysis software (version 5.01). In regard to pseudovirus neutralization, the criterion for a positive response was 3-fold above the observed background in the pre-bleed sample.

#### ELISPOT

The mean and standard deviation of the assay spot count from triplicate wells were reported. The Student t test was used to compare the immune response between test wells and wells receiving ‘no antigen’. A *p* value of ≤ 0.05 was used as the indication of a significant response. Additionally, to be considered a positive response, a minimum of 50 IFNγ SFC had to be achieved. 

## Results

### Ad4Env120, Ad4Env140, and Ad4Env160 Recombinant Virus Generation

The Ad4Env120, Ad4Env140, and Ad4Env160 viruses were generated in A549 cells. Extraction of the viral DNA and restriction digestion mapping with several combinations of enzymes confirmed that the viral genome was intact without major deletions or insertions (data not shown). PCR amplification across the E3 region, including the expression cassette, generated a DNA product of the correct size and sequencing of the PCR product confirmed the sequence identity of the transgene and flanking regions (data not shown).

### Ad4 env Transgene Expression of gp120, gp140, and gp160 in A549 Cells

**Figure 2:**
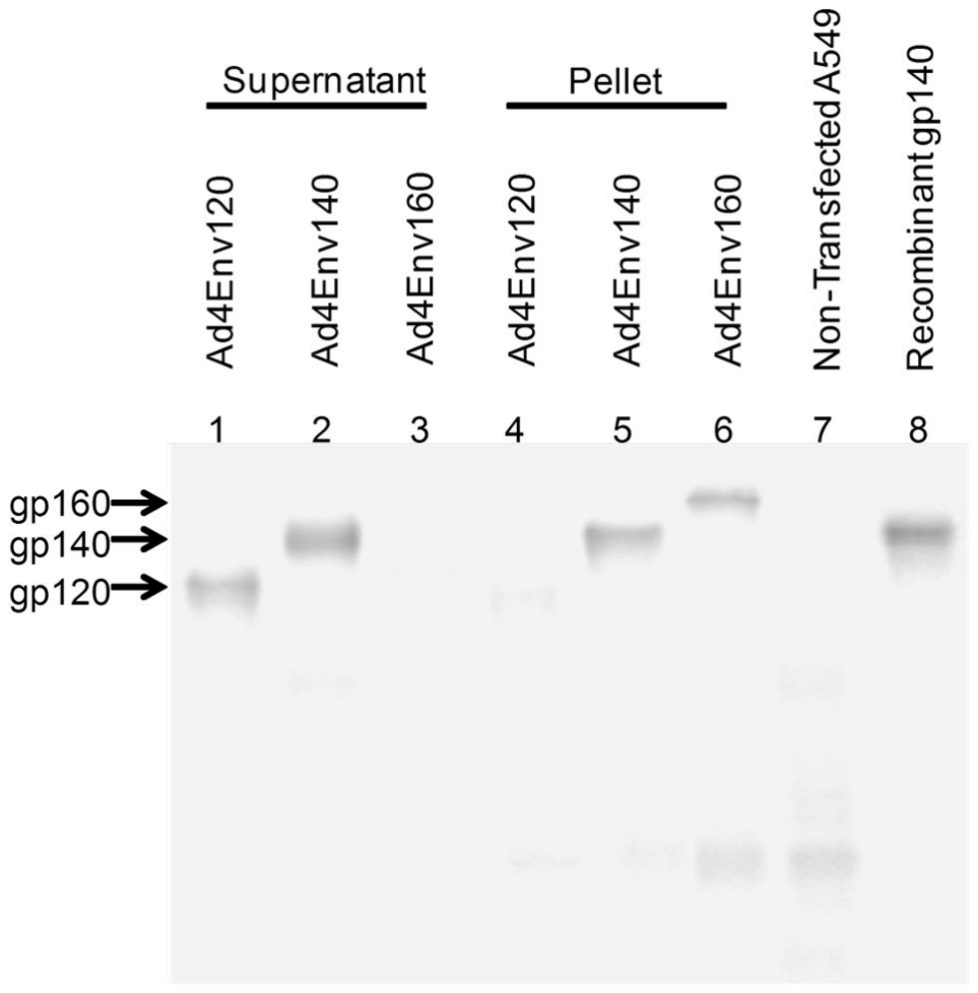
Western blot analysis of env transgene protein expression. A549 cells were infected with Ad4Env120, Ad4Env140 or Ad4Env160 recombinant viruses and harvested two to three days later. Comparable amounts of each Env protein (approximately 2 µg) were run on a SDS-PAGE gel and transferred to nitrocellulose. Env proteins were labelled with a mouse anti-HIV Env 1086 clade C (VRC-C 3B3) antibody which was then detected with a goat anti-mouse HRP antibody.

Env glycoprotein expression and secretion were evaluated by western blot analysis after infection of A549 cells with Ad4Env120, Ad4Env140, or Ad4Env160 recombinant vectors (Figure **2**). As expected, Env gp120 and gp160 expressed glycoproteins were detected largely in the supernatant or cell pellet fractions, lanes 1 and 6, respectively. The Env gp140 expressed glycoprotein was associated with both-supernatant and cell pellet, lanes 2 and 5, respectively. Recombinant Env gp140 was used as a positive control, lane 8, which corresponded in size to the gp140 expressed from the Ad4Env140 vector.

### Cell Surface Recognition of Env160 and Env160K→N Glycoproteins by bNAbs

**Figure 3:**
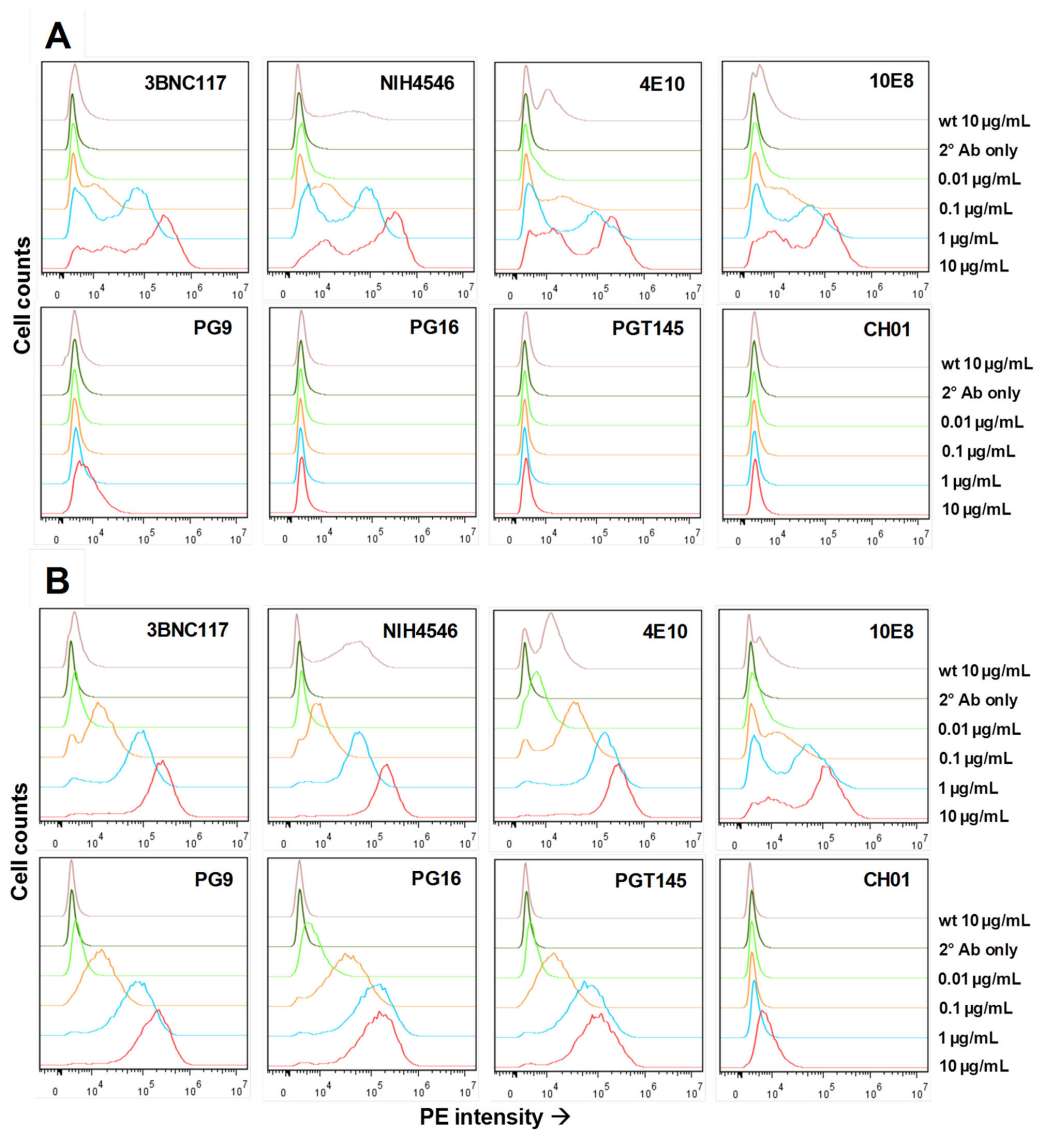
Recognition of cell surface Env 1086 clade C glycoprotein by bNAbs. A549 cells infected at a concentration 5 × 10^8^ vp/mL of Ad4Env160, (A) and Ad4Env160K→N (B). Cells were harvested 18 hours after infection and flow cytometry performed using 10, 1.0, 0.1, and 0.01 µg/mL concentrations of bNAb as primary and goat anti-human R-PE as secondary antibody. The reactivity of each bNAb at the highest concentration against the negative control Ad4wt-infected cells is shown in each panel (indicated by “wt 10 µg/mL”). Any non-specific background staining shown was not evident at lower concentrations of bNAbs. Binding of the secondary antibody alone is indicated by “2° Ab only”.

A panel of bNAbs recognizing various regions of the Env glycoprotein including: 1) CD4 binding site (CD4bs); 2) membrane proximal external region (MPER); 3) V1V2 loops; and 4) V3 loop were used to evaluate the antigenicity of cell surface-expressed Env gp160 and Env gp160K→N. A549 cells were infected with Ad4Env160 recombinant virus, stained with bNAbs, and evaluated for cell surface Env160 glycoprotein expression using flow cytometry. Env gp160 expressed on the surface of A549 cells was recognized by CD4bs-specific antibodies, including 3BNC117 and NIH 45-46, and the MPER-specific antibodies 4E10 and 10E8 (Figure **3A**). Recognition of Env gp160 was evident by peak shift when using 0.1 µg/mL and higher concentrations of bNAbs. However, conformation-dependent antibodies (PG9, PG16, PGT145, and CH01) recognizing V1V2 loops were not reactive, even at 10 µg/mL, the highest concentration of bNAb used. A single amino acid in the 1086 clade C Env glycoprotein at position 160 (HΧB2 numbering sequence) in the V2 loop was changed by site-directed mutagenesis from lysine (K) to asparagine (N) to enable N-linked glycosylation at this site [37]. The recombinant Ad4 vector expressing Env160K→N was then used to infect A549 cells, and surface expression of the mutated Env glycoprotein was now clearly recognized by PG9, PG16, and PG145 bNAbs (but not CH01) at concentrations of 0.1 µg/mL and higher (Figure **3B**). The mutagenesis did not affect the binding of CD4bs and MPER-specific bNAbs which retained reactivity for the Env160K→N glycoprotein. A total of 19 bNAbs were used to evaluate the antigenicity of Env gp160 and Env gp160K→N (Table **1**). A majority of the bNAbs recognized both Env gp160 and Env gp160K→N suggesting that both Env proteins were presented on the infected cell surface in an appropriate conformation. bNAb reactivity, particularly at lower antibody concentrations, was not observed when the cells were infected with Ad4wt virus or when only secondary antibody was used.

**Table 1:** bNAb Binding to Surface-Expressed HIV-1 Env gp160.

**bNAb**	**Year of Discovery**	**Env Glycoprotein**	**Target Site**	**Env160**	**Env160 K→N**
**NIH45-46**	2011	gp120	CD4-binding site	+++	++
**NIH45-46 G54W**	2011	gp120	CD4-binding site	+++	++
**3BNC60**	2011	gp120	CD4-binding site	+++	+++
**3BNC117**	2011	gp120	CD4-binding site	+++	+++
**b12**	1994	gp120	CD4-binding site	+++	+++
**CH 31**	2011	gp120	CD4-binding site	+	+
**PG9**	2009	gp120	V1/V2 loops	+/-	+++
**PG16**	2009	gp120	V1/V2 loops	-	+++
**PGT145**	2011	gp120	V1/V2 loops	-	+++
**CH01**	2010	gp120	V1/V2 loops	-	+/-
**PGT121**	2011	gp120	Glycan/V3 loop	+++	+++
**PGT123**	2011	gp120	Glycan/V3 loop	++	+++
**PGT126**	2011	gp120	Glycan/V3 loop	+	+
**PGT128**	2011	gp120	Glycan/V3 loop	+/-	+
**2G12**	1994	gp120	Glycan	+	+
**2F5**	1993	gp41	MPER	-	-
**4E10**	1994	gp41	MPER	++	+++
**CH 12**	2011	gp41	MPER	++	++
**10E8**	2012	gp41	MPER	+++	++

Symbols representing bNAb binding to Env gp160 are depicted as:

^+++^ = positive high binding as indicated by a histogram shift at 10, 1, and 0.1 µg/mL

^++^ = medium binding, shifts at 10 and 1 µg/mL

^+^ = low binding, shift at 10 µg/mL only

^+/-^ = weak binding at 10 µg/mL

^-^ = no binding

### Ad4Env120, Ad4Env140, and Ad4Env160 Recombinant Virus Production Yields

Ad4Env recombinant viruses were produced and purified to a scale sufficient for use in small animal immunogenicity studies (Table **2**). It should be noted that the average purified recombinant virus yield per cell stack (CS) was comparable for Ad4Env120 and Ad4Env140 viruses, 1.38 × 10^13^ vp and 1.81 × 10^13^ vp, respectively. In contrast, recombinant virus yield per cell stack for Ad4Env160 virus was approximately 11-fold less, 1.44 × 10^12^ vp. The lower yield of virus may indicate toxic effects on the cell substrate when expressing the full-length Env gp160.

**Table 2:** Productivity of Ad4Env Recombinant Viruses in A549 Cells.

**Vector Name**	**Infection (vp/mL)**	**CellStacks®**	**Total Yield**	**Yield/C S**
**Ad4Env160**	1 × 10^8^	8	1.16 × 10^13^	1.44 × 10^12^
**Ad4Env140**	1 × 10^8^	2	3.63 × 10^13^	1.81 × 10^13^
**Ad4Env120**	1 × 10^8^	2	2.76 × 10^13^	1.38 × 10^13^

### Ad4Env-Induced Humoral Responses in Rabbits

**Figure 4:**
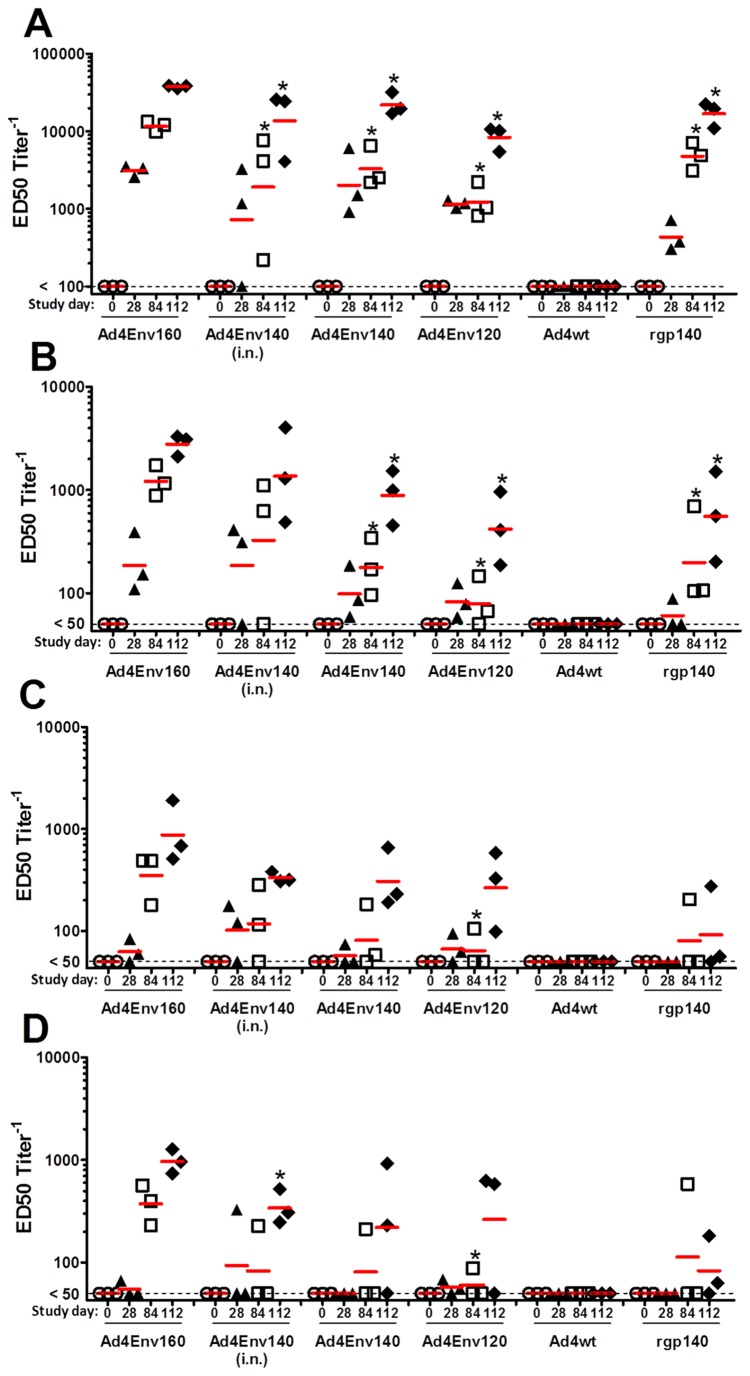
Immunogenicity of Ad4Env160, Ad4Env140, and Ad4Env120 recombinant viruses in rabbits. Three rabbits per group were immunized on days 0 and 28 with each of the Ad4Env 1086 clade C recombinant vectors and booster immunizations were performed on day 84 with recombinant Env 1086 clade C 140 glycoprotein. Ad4Env vectors were administered by the i.m route with the exception of one group which also received the Ad4Env140 vector by the i.n. route. Positive control rabbits were immunized three times with the recombinant Env 1086 clade C gp140 in Rehydragel on days 0, 28 and 84. Negative control rabbits were immunized three times with Ad4wt virus, i.m., on days 0, 28 and 84; antibody titers were all <50 for this group. Serum samples were collected on days 0 (pre-vaccine), 28, 84 and 112. The ELISA coating antigens were: (A) Env 1086 clade C 140 glycoprotein; (B) Env140 1086 clade C V1V2 polypeptide; (C) Env A244 AE strain V1V2 polypeptide; and (D) clade B Env CASE A V1V2 scaffold fusion protein. For each of the ELISA antigens, statistical analysis using the t-test was performed comparing antibody titers induced by the Ad4Env160 vector versus titers induced by each of the other Ad4Env vectors or by the Env140 1086 clade C glycoprotein. Groups with a statistical difference (*p* ≤ 0.05) in geomean ED_50_ antibody titers relative to Ad4Env160 vector immunogen are denoted by an asterisk.

Ad4 recombinant vectors expressing Env gp120, Env gp140, and Env gp160 glycoproteins were used to immunize rabbits. Rabbits were immunized twice on days 0 and 28 with the Ad4Env recombinant viruses, followed by a recombinant Env gp140 1086 clade C booster immunization formulated in Rehydragel® on day 84 (two months after the last recombinant vector immunization). Blood was collected on days 0, 28, 84, 112 and then evaluated by ELISA for binding antibodies to Env gp140 (1086 clade C) and Env V1V2 antigens (1086 clade C, AE A244 clade AE (clade E Env), and gp70 V1V2 CASE A2 clade B fusion protein, clade B). Note that all groups received the recombinant Ad4Env vectors by the intramuscular (i.m.) route with the exception of a single group which was immunized with the Ad4Env140 vector by the intranasal (i.n.) route. High levels of Env gp140-specific antibodies were induced in the rabbits following two recombinant vector immunizations; geomean ED_50_ antibody titers, ranging from 1,229 to 11,618, (Day 84, Figure **4A**). Following the Env gp140 boost immunization (Day112), increased geomean ED_50_ antibody titers in the range of approximately 8,317 to 37,473 were elicited. In general, after two immunizations, the Ad4 vector expressing full-length Env160 was more immunogenic than comparable vectors expressing Env140 or Env120 glycoproteins: Ad4Env160 (11,618 ED_50_) vs. Ad4Env140 i.n. (1,906 ED_50_, *p* ≤ 0.03); Ad4Env140 (3,293 ED_50_, *p* ≤ 0.01); and Ad4Env120 (1,350 ED_50_, *p* ≤ 0.001). A similar pattern was evident after the booster immunizations with the Env gp140 protein: Ad4Env160 (37,473 ED_50_) vs. Ad4Env140 i.n. (13,649 ED_50_, *p* ≤ 0.05); Ad4Env140 (21,876 ED_50_, *p* ≤ 0.03); and Ad4Env120 (8,317 ED_50_, *p* ≤ 0.0001). Ad4Env160 given twice and followed by the Env gp140 boost immunization (37,473 ED_50_) was significantly more immunogenic vs. protein given 3 times (16,840 ED_50_, *p* ≤ 0.005), respectively. As expected, Ad4wt virus did not induce Env gp140-specific antibodies. 

The higher immunogenicity of Ad4 vectors expressing Env gp160 relative to gp140 and gp120 was also evident when evaluating geomean ED_50_ antibody titers specific for Env gp120 V1V2 antigens (Figure **4B-D**). Please note the maximum scale of ED_50_ antibody titer is 10,000 for Figure **4B-D**. In regard to binding antibodies specific for 1086 clade C V1V2 polypeptide, Ad4Env160 vector was significantly more immunogenic than Ad4Env140 and Ad4Env120 vectors after two recombinant vector immunizations (day 84) and this difference was maintained after the recombinant Env gp140 booster immunization (day 112) (Figure **4B**). The following geomean ED_50_ antibody titers were induced by day 84: Ad4Env160 (1,213) vs. Ad4Env140 i.n. (326); Ad4Env140 (177, *p* ≤ 0.015); Ad4Env120 (79, *p* ≤ 0.009); and Env gp140 protein (198, *p* ≤ 0.04). Following Env gp140 boost immunization (day 112), Ad4Env160 (2,784) was again the most potent prime immunogen: vs. Ad4Env140 i.n. (1,365); Ad4Env140 (883, *p* ≤ 0.02); Ad4Env120 (417, *p* ≤ 0.006); and Env gp140 protein (554, *p* ≤ 0.017).

Also noted, were the differences in Ad4Env immunogenicity when evaluating induction of cross-reactive geomean ED_50_ antibody titers against the clade AE (Figure **4C**) and clade B (Figure **4D**) V1V2 antigens. In both cases, cross clade-reactive binding antibodies were more readily induced using the Ad4Env160 immunogen. Specifically, in the case of reactivity to AE A244 V1V2 polypeptide, the geomean ED_50_ antibody titers induced with the Ad4Env160 prime and Env gp140 1086 clade C booster immunizations were 874 on day 112 relative to 333 for the Ad4Env140 vector delivered i.n., 306 for the Ad4Env140 vector and 265 for the Ad4Env120 vector (Figure **4C**). Recombinant Env gp140 formulated in Rehydragel® and given three times induced V1V2-specific ED_50_ antibody titer of 274 in only one of three rabbits. The superiority of Ad4Env160 as an immunogen was also evident in the case of cross-reactive V1V2-specific antibodies to the gp70 V1V2 CASE A2 clade B fusion protein (Figure **4D**). Specifically, an Ad4Env160 recombinant virus prime followed by an Env gp140 boost (day 112) were capable of inducing geomean ED_50_ antibody titers specific for clade B V1V2 fusion protein of 964. Additionally, Ad4Env160 vector given twice induced geomean ED_50_ antibody titers of 373 prior to the Env gp140 boost immunization. In contrast, using Ad4Env140 i.n., Ad4Env140, and Ad4Env120 immunogens as the priming immunization, followed by the Env gp140 1086 clade C booster immunization resulted in mean ED_50_ antibody titers of 341, 220, and 264, respectively. Recombinant Env gp140 formulated in Rehydragel® given two or three times induced cross-reactive ED_50_ antibody titers of 583 and 182 but in only one of three rabbits. Of note, especially in the case of cross clade-reactive V1V2 binding antibodies (Figure **4C and 4D**), the Ad4Env160 vector given twice induced significant responses in three of three rabbits vs. responses in only one or two rabbits when immunized with the Ad4Env140 and Ad4Env120 vectors.

In summary, full-length Env160 expressed from recombinant Ad4 vector was a more potent immunogen than either Ad4Env140 or Ad4Env120 in terms of inducing overall binding antibodies to Env gp140 and against the homologous Env V1V2 loop epitopes, and this difference was also observed in the induction of antibodies to both the clade AE- and B-derived V1V2 antigens.

The rabbit immune sera were also evaluated for neutralization of tier 1 and 2 viruses. As shown in Table **3**, recombinant Ad4 vectors expressing Env gp160, gp140 and gp120 as the prime immunization followed by Env gp140 (1086 clade C) as the boost immunization induced neutralizing antibody titers specific for MW965.26, a tier 1 clade C virus. Significant differences in immunogenicity induced was evident following two recombinant Ad4Env virus immunizations (day 84): geomean ID_50_ antibody titers were Ad4Env160 (2,868) vs. Ad4Env140 i.n. (171, *p* ≤ 0.028); Ad4Env140 (685, *p* ≤ 0.04); and Ad4Env120 (230, *p* ≤ 0.02). The ID_50_ antibody titers increased considerably following Env gp140 protein boost immunization, although differences between vector groups were not significant: geomean ID_50_ antibody titers were Ad4Env160 (11,133), Ad4Env140 i.n. (1,541); Ad4Env140 (2,654); and Ad4Env120 (2,036). In contrast, low but significant neutralization of the homologous Ce1086_B2.LucR.T2A.ecto tier 2 clade C virus was most evident with the recombinant Ad4Env140 vector delivered by either the i.n. or i.m. routes. Following two Ad4Env vector or Env gp140 immunizations (day 84), the geomean ID_50_ antibody titer values were the following: Ad4Env160 (22); Ad4Env140 i.n. (71); Ad4Env140 (50); Ad4Env120 (40); and Env gp140 (38). Rabbits immunized with the Ad4wt virus, as expected, did not induce immune sera capable of neutralizing the Ce1086 tier 2 virus. The Ad4Env140 vaccine also induced, in three of three rabbits, low but significant neutralization of the heterologous clade C Du151.2.LucR.T2A.ecto virus in the range of 37 to 69 (geomean, 46) ID_50_ antibody titer following two vector immunizations, day 84. The other Ad4Env vectors and Env gp140 protein also induced neutralization in the same range but the neutralization was less consistent and typically observed for only one or two rabbit immune sera. The rabbit immune sera were evaluated for neutralization activity against three additional tier 2 clade C viruses; Ce1176.LucR.T2A.ecto, Ce2010.LucR.T2A.ecto, and Du422.1.LucR.T2A.ecto. Neutralization of Ce1176.LucR.T2A.ecto virus was not detected (data not shown). However, sporadic neutralization of Ce2010.LucR.T2A.ecto, and Du422.1.LucR.T2A.ecto viruses, in the range of 40 to 99 ID_50_ antibody titer, was observed following two Ad4Env vector (Env140 or Env120) immunizations and an Env gp140 boost immunization but typically for only one of three rabbits (data not shown). Additionally, Ce2010.LucR.T2A.ecto and Du422.1.LucR.T2A.ecto tier 2 virus neutralization was not observed when immune sera were induced with either Ad4Env160 vector prime immunization followed by Env gp 140 boost immunization or Env gp140 given three times (data not shown).

**Table 3:** Neutralization of Tier 1 and 2 Clade C Viruses.

		MW965.26^a)^				Ce1086_B2.LucR.T2A.ecto^b)^				Du151.2.LucR.T2A.ecto^c)^				
		**Day**				**Day**				**Day**				
**Immunogen**	**Rabbit #**	**0**	**28**	**84**	**112**	**0**	**28**	**84**	**112**	**0**	**28**	**84**	**112**	
**Ad4Env160**	1	--^d)^	**37** ^e)^	**2964**	**5250**	--	**22**	--	--	23	25	22	32	
	2	--	**45**	**1771**	**10308**	--	--	**24**	**29**	--	**22**	**38**	**24**	
	3	--	**92**	**4493**	**25501**	--	**27**	**21**	**37**	26	48	24	30	
**Ad4Env140 (i.n.)**	1	--	**27**	**399**	**5014**	--	--	**34**	**117**	23	23	35	**105**	
	2	--	**30**	**624**	**4831**	--	**77**	**413**	**923**	74	23	39	83	
	3	--	--	--	**151**	--	--	**25**	**56**	44	27	--	99	
**Ad4Env140**	1	--	--	**535**	**2480**	--	--	**29**	**39**	--	**29**	**39**	**76**	
	2	--	**235**	**1105**	**3773**	--	--	**108**	**488**	--	**22**	**37**	**34**	
	3	--	**24**	**534**	**1998**	--	**26**	**40**	**74**	--	**23**	**69**	**38**	
**Ad4Env120**	1	--	--	**115**	**1758**	--	--	**47**	**47**	28	34	38	39	
	2	--	**33**	**452**	**2927**	--	**25**	**43**	**111**	--	**23**	**25**	**26**	
	3	--	**42**	**110**	**1641**	--	**21**	**32**	**26**	20	34	34	45	
**Ad4wt**	1	--	--	--	--	--	--	--	--	--	--	--	--	
	2	--	--	--	**33**	--	--	--	--	--	**33**	--	--	
	3	--	--	--	--	--	--	--	--	--	--	--	--	
**Env gp140**	1	--	**25**	**486**	**2766**	--	**37**	**58**	**34**	--	**21**	**22**	**26**	
	2	--	--	**235**	**1742**	--	--	**34**	**31**	--	**21**	--	--	
	3	--	--	**205**	**810**	--	--	**28**	--	--	--	**25**	**28**	

^a)^ Tier 1 clade C pseudovirus

^b)^ Tier 2 clade C homologous infectious Renilla luciferase-expressing reporter virus

^c)^ Tier 2 clade C heterologous infectious Renilla luciferase-expressing reporter virus

^d)^ '--' = <20, ID50 antibody titer

Values are the serum dilution at which relative luminescence units (RLUs) were reduced 50% vs. virus control wells (no test sample)

^e)^ Values in bold type scored positive for neutralization based on the criterion of >3 times the observed background in the pre-bleed (day 0)

### Ad4Env160K→N-Induced Env-Specific Cellular Responses in Mice

**Figure 5:**
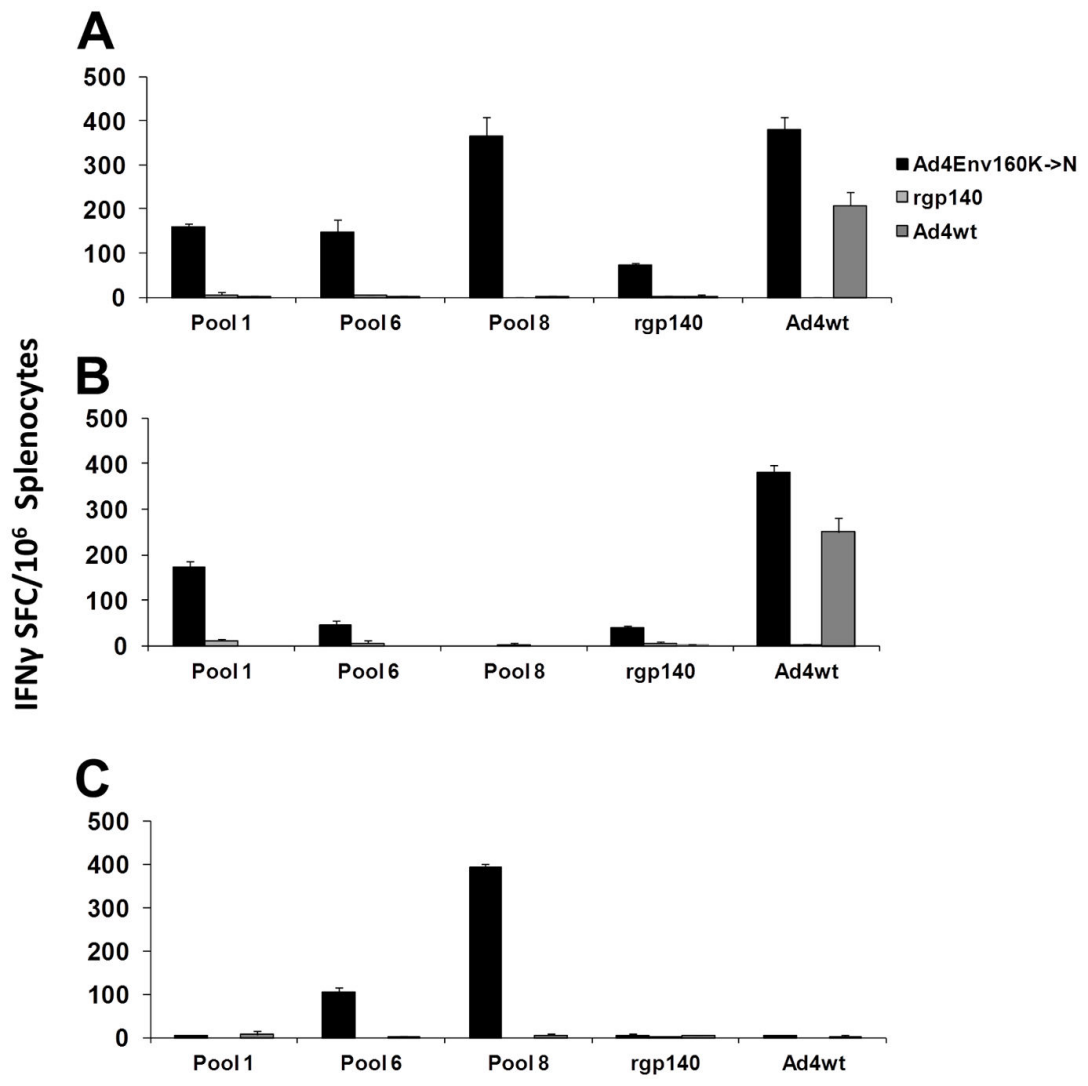
Ad4Env160K→N vector-induced Env-specific cell-mediated immunity in mice. C57BL/6 × BALB/c F1 (CB6F1) mice (6 animals/group) were immunized two times, days 0 and 28, with Ad4Env160K→N recombinant virus, Ad4wt virus, and recombinant Env gp140 (rgp140). On day 56 the mice were sacrificed and splenocytes pooled and tested in an IFNγ ELISPOT. Whole splenocytes (A), CD4^+^ (B), and CD8^+^ (C) T cells were evaluated. 15-mer peptides from the HIV-1 consensus clade C Env gp160 sequence, Env gp140 1086 clade C protein, and heat denatured Ad4wt virus were used as target antigens.

Groups of mice were immunized two times with either, the recombinant Ad4Env160K→N virus, Ad4wt virus, or recombinant Env gp140 formulated in MPL/Rehydragel®. Splenocytes were obtained for evaluating cellular immunity using an IFNγ ELISPOT assay and overlapping Env (consensus clade C) peptide sets, recombinant Env gp140, and heat-inactivated Ad4wt virus as antigens. Following Ad4Env160K→N vector immunization, significant Env-specific cellular responses were induced to several regions of the Env molecule as denoted by reactivity to peptide pools 1, 6, and 8 in the range of 150 to 370 IFNγ SFC per 1 × 10^6^ whole splenocytes (Figure **5A**). Pools 1, 6, and 8 contain peptides from the gp120, gp41, and cytoplasmic tail regions of Env glycoprotein, respectively. When specific T cell populations were depleted from total splenocytes, pool 1 responses were shown to be primarily CD4^+^ T cell-specific (Figure **5B**) while pools 6 and 8 were primarily CD8^+^ T cell-specific (Figure **5C**). Ad4Env160K→N and Ad4wt viruses induced primarily vector-specific CD4^+^ T cells (Figure **5B**). The recombinant Env gp140 immunogen (rgp140) did not induce significant T cell responses.

## Discussion

An HIV-1 vaccine strategy that induces durable, protective humoral and cellular immunity has yet to be developed. To address this goal, we have built on the modest success of the RV144 Thai trial and our clinical experience with the Ad4-H5-Vtn influenza vector [25] to evaluate a heterologous prime-boost strategy for an HIV-1 vaccine consisting of an oral replication-competent Ad4-HIV-1-Env recombinant vector prime followed by a recombinant Env glycoprotein boost. Our hypothesis is that for induction of a protective immune response, an Env-based HIV-1 vaccine is required, along with a Gag-based internal protein component. Furthermore, the prolonged intracellular presentation in GI mucosal cells through delivery by an oral replicating vector followed by systemic protein boosting is a logical scientific approach. The safety and immunogenicity of the replication-competent, orally administered Ad4 vector technology has recently been documented in a H5 influenza Phase 1 study, and this system may provide a potential means to express and present on the cell membrane the correct conformation of Env glycoprotein required for induction of protective immune responses. It should be noted that the human Ad4 vaccine vectors expressing HIV-1 Env described in this paper do not carry out productive infections in cells of nonhuman origin but, similar to defective Ad5 vectors, they will infect cells from multiple species including mice, rabbits, and many species of NHPs, and these cells will effectively support transcription and translation of both Ad viral proteins and foreign transgenes. The full immunological benefits of the Ad4 replication-competent vector as a vaccine can only be evaluated in humans where virus replication would likely result in infection of more cell types and amplification of the amount of transgene protein delivered to the immune system.

In this study, we generated Ad4 recombinant constructs encoding HIV-1 Env 1086 clade C glycoproteins. Following infection of A549 cells in vitro, env transgene protein expression was confirmed by western blot and recognition of cell surface Env160 glycoprotein by bNAbs. Interestingly, we were able to acquire additional bNAb reactivity by engineering a specific amino acid change (K→N at position 160) in the Env gp120 V2 sequence. This amino acid change enabled binding by conformation-dependent bNAbs, PG9, PG16, and PG145. Whether increased Env glycoprotein antigenicity will translate into augmented immunogenicity in vivo in humans is not known. Immunogenicity of the recombinant Ad4Env vectors was demonstrated by induction of Env-specific antibody and cellular responses in rabbits and mice, respectively. Several key aspects of vaccine immunogenicity were observed during the study: 1) magnitude of Env-specific humoral immunogenicity was generally dependent on env transgene length with gp160> gp140> gp120; 2) antibodies specific for V1V2 peptide were induced; and 3) induction of Env-specific cellular immunity was also accomplished. In regard to induction of cellular immunity, it should be noted that we evaluated responses following recombinant Ad4Env vector immunization only. It is not known whether a recombinant Env protein boost immunization would have augmented the immune responses and/or changed the CD4^+^ and CD8^+^ T cell profile. Interestingly, in the case of tier 2 virus neutralization, the AdEnv140 vector was more immunogenic than the Ad4 vector expressing the full-length Env 160 glycoprotein. This is in contrast to neutralization of the tier 1 MW965.26 pseudovirus where the Ad4Env160 vector was the superior immunogen. Additional Ad4Env constructs are being generated to follow-up on this observation and generate vaccines that potentially improve upon neutralization of tier 2 viruses. In addition to Env length, several other variables will be addressed, individually and in various combinations, regarding their potential to improve Env-specific antibody responses including: 1) state of furin cleavage site, i.e., mutated to prevent cleavage vs. wild type; 2) exogenous vs. endogenous promoters to drive Env transgene expression; and 3) K→N mutation at position 160 in the V2 loop vs. wild type.

These studies would suggest that development of a vaccine product expressing a membrane-bound full-length Env gp160 may be preferable vs. secreted gp140 or gp120 in regard to induction of Env-specific antibodies. In regard to cellular immunity, robust Env-specific T cell responses were elicited following immunization of mice with the Ad4Env160K→N vector. Significant IFNγ responses were induced, in part, to the cytoplasmic tail (CT) (pool 8) and these responses may not be realized using either Ad4Env140 or Ad4Env120 vaccines which lack the CT. Whether the CT-specific T cell responses will have an added benefit above T cell responses that are induced specific for gp140 or gp120 is not known. The challenge of advancing the Ad4Env160 vaccine was witnessed in the context of virus production where Ad4Env160 was approximately 10-fold less productive vs. AdEnv140 and Ad4Env120 viruses. Diminished virus yield may be attributed to cytopathic effects of the Env cytoplasmic tail region [38-41]. In studies outside the scope of this report, we generated recombinant Ad4 vectors expressing Env with truncations of the cytoplasmic tail but still retain the transmembrane region. These Ad4 recombinant vectors express membrane bound gp150 (40 amino acids of CT remain; 118 amino acids were deleted) and gp145 (4 amino acids of CT remain; 154 amino acids were deleted) (Alexander et al., unpublished). The Ad4Env150 and Ad4Env145 recombinant viruses were approximately 5-fold more productive in virus yield vs. Ad4Env160 virus in A549 cells and thus provide an attractive path for Ad4Env vaccine development (Alexander et al., unpublished). Potentially, Ad4Env160 vaccine yield could be improved by suppressing Env160 transgene expression during vaccine production. Wang demonstrated that the yield of adenoviral vector particles was increased 10-fold in a packaging cell line with stable production of a short hairpin RNA (shRNA) that can silence the transgene vs. a cell substrate not expressing shRNA [42]. 

Of note was the capacity of the Ad4Env vaccines to induce V1V2 peptide-specific binding antibodies to clades C, AE, and B. This immunogenicity characteristic is likely to be important for HIV-1 vaccine development, in part, due to the identification of V1V2 peptide-specific antibodies as a correlate of decreased transmission risk in the RV144 study [11,43,44]. The V1V2 loop has a number of features that may be important during initial events of virus infection. It has a highly conserved length among early (transmitted/founder) isolates and escape from neutralizing antibodies is associated with increased V1V2 length and glycosylation [45,46]. Also, V2 loop contains a potential α4β7 integrin-binding motif that may promote infection of lymphocytes localized at mucosal tissues [47,48]. Karasavvas et al., using peptide microarray analysis, ELISA, and Biacore analyses, demonstrated that the immunogens used in the RV144 trial induced antibodies to the V2 loop of gp120 from diverse HIV-1 subtypes [43]. In addition, from analysis of RV144 samples, Rolland et al., reported that mutations that select against, or sieve HIV-1 breakthrough infections contain mutations at positions 169 and 181 in V2 [44]. Several other investigators have indentified the V1V2 loop as a target for bNAbs such as PG9 and PGT145 that engage V1V2 and neutralize approximately 80% of HIV-1 isolates [37,49,50]. Taken together, these studies suggest that vaccines designed to induce V1V2-specific antibodies higher than observed in the RV144 trial may have better potential to protect against HIV-1 infection [11].

It is generally thought that a HIV-1 vaccine based in part on a neutralizing antibody response would be beneficial [51,52]. In the study reported herein, neutralization of the tier 1 clade C virus, MW965.26, was readily accomplished with the Ad4Env vectors. Lower, but significant, neutralization of homologous tier 2, clade C (Ce1086_B2.LucR.T2A.ecto) and heterologous tier 2, clade C (Du151.2.LucR.TwA.ecto) viruses was also observed. It has been difficult to elicit potent and cross-reactive neutralizing antibody responses by immunization with Env-based vaccine candidates [53]. This difficulty may be due to immune dominance of gp120 variable regions, glycan occlusion, and/or inability of germline versions of the bNAbs to bind Env [50,54-59]. Thus, vaccine efforts to date have failed to address the tier 2 phenotype [36,51,52]. Neutralization of several tier 1 viruses was detected in the RV144 trial, but not tier 2 viruses, which suggest that modest efficacy observed in the trial was mediated by other immune responses, either alone or in combination with tier 1 neutralizing antibodies [35]. To improve upon vaccine induction of more potent broadly neutralizing antibodies, several approaches have been suggested including: 1) B-cell-lineage vaccine design [60-62]; and 2) structure-guided vaccine design [53,63-66].

Of interest in regard to vaccine development, is the effect of pre-existing Ad4 immunity on vaccine potency. This is relevant since the overall prevalence for Ad4 neutralizing antibodies is approximately 30% among unimmunized US Army Trainees [67]. We evaluated the effects of pre-existing Ad4 immunity in a Phase 1 trial where subjects (approximately 30% with pre-existing Ad4 neutralizing antibody) received a recombinant Ad4 vaccine expressing avian influenza H5 hemagglutinin [25]. At high vaccine doses of 10^9^-10^11^ viral particles (10^7^-10^9^ infectious units), hemagglutinin-specific cellular responses and priming for HAI antibody responses were comparable in Ad4 seronegative and seropositive subjects. Thus, these results suggest that higher doses of vaccine may overcome the effects of pre-existing Ad4 immunity.

Ad4 recombinant vectors expressing HIV-1 (Env and mosaic Gag) immunogens will be evaluated in a Phase 1 safety and immunogenicity trial beginning in October, 2013. Together with the completed Ad4-H5-Vtn influenza Phase 1 trial these data may potentially support use of an Ad4 replicating vector system for use in conjunction with newly developed HIV-1 immunogens.

In summary, we have evaluated several pre-clinical properties of the Ad4Env vector vaccine. These data support further evaluation of these new vaccines and if warranted, Phase 1 clinical trial evaluation.
